# Supplementary material for: Immune Response Gaps Linked to SARS-CoV-2 Infection: Cellular Exhaustion, Senescence, or Both?
Source: Int J Mol Sci. 2022 Nov 8;23(22):13734. doi: 10.3390/ijms232213734 (PMC9696576; doi:10.3390/ijms232213734)
Supplement: Supplementary file 1 [file ijms-23-13734-s001.zip › ijms-1936958-supplementary.pdf]

Supplementary Table S1: Primary antibody data.

Antibody	Type	Clone/Code	Dilution	Source
Anti-Arginase	Monoclonal/Rabbit	E-P261	1:200	BioSB
Anti-CD3	Polyclonal/Rabbit	IR503	Ready-to-use	Dako
Anti-CD4	Monoclonal/Rabbit	4B12	Ready-to-use	Dako
Anti-CD8	Monoclonal/Rabbit	C8/144B	Ready-to-use	Dako
Anti-CD20	Monoclonal/Mouse	IR604	Ready-to-use	Dako
Anti-CD57	Monoclonal/Mouse	IR647	Ready-to-use	Dako
Anti-CD68	Monoclonal/Mouse	KP-1	1:400	BioSB
Anti-CCR4	Polyclonal/Rabbit	Ab216560	1:100	Abcam
Anti-IL-4	Polyclonal/Rabbit	PA5 25165	1:200	Thermo Fisher
Anti-INF-α	Polyclonal/Rabbit	A0285	1:200	ABclonal
Anti-INF-γ	Polyclonal/Rabbit	Ab218426	1:100	Abcam
Anti-iNOS	Monoclonal/Rabbit	E-P261	1:100	BioSB
Anti-Perforin	Monoclonal/Mouse	5B10	1:200	BioSB
Anti-Sphingosine-1	Polyclonal/Rabbit	Ab71700	1:200	Abcam

Supplementary Table S2: Clinical data regarding Comorbidities, Computed tomography chest at admission, Relevant initial laboratory tests, Laboratory tests 24 hours before death and Therapeutic drugs.

	CASE 1	CASE 2	CASE 3	CASE 4	CASE 6	CASE 7
**Gender / Age**	Female, 87 years old	Male, 53 years old	Female, 85 years old	Male, 73 years old	Male, 80 years old	Male, 81 years old
**Comorbidities**	Systemic Arterial Hypertension<br>Dyslipidemia<br>Hypothyroidism<br>Senile dementia	Class II obesity	Systemic Arterial Hypertension<br>Brain Stroke<br>Vascular Dementia	Type 2 Diabetes Mellitus<br>Chronic Kidney Disease Dialysis<br>Atrial Fibrillation<br>Coronary Disease<br>Heart Failure<br>Peripheral Obstructive Artery Disease	Systemic Arterial Hypertension<br>Coronary Disease<br>Heart Failure<br>Bronchial Asthma<br>Class III obesity	Systemic Arterial Hypertension<br>Chronic Kidney Disease Dialysis<br>Brain Stroke
**Computed tomography chest at admission**	Diffuse and bilateral "opacities with ground-glass attenuation," thickening of the pulmonary septum, suggestive of viral pulmonary infection	Diffuse and bilateral "opacities with ground-glass attenuation" suggestive of viral pulmonary infection	Image of "opacities with ground-glass attenuation" upper left lobe, suggestive of viral pulmonary infection and small volume pleural effusion	Diffuse and bilateral "opacities with ground-glass attenuation", suggestive of viral pulmonary infection	Diffuse and bilateral "opacities with ground-glass attenuation", suggestive of viral pulmonary infection	Diffuse and bilateral "opacities with ground-glass attenuation", suggestive of viral pulmonary infection
**Relevant initial laboratory tests**	- C Reactive Protein=313mg/L<br>- Globular volume=24.8%<br>- Hemoglobin=8.7g/dL<br>- Total leukocytes=15,100/ band cells=1208 (8%) and lymphocytes=604 (4%)<br>- Blood platelets=366,000	- C-Reactive Protein=146mg/L<br>- Globular volume=38.1%<br>- Hemoglobin=13.5g/dL<br>- Total leukocytes=11,000/band cells=550 (5%) and lymphocytes=990 (9%)<br>- Blood platelets=325,000	- C-Reactive Protein=393mg /L;<br>- Globular volume=28.7%<br>- Hemoglobin=9.9g/dL<br>- Total leukocytes=9,900/band cells=396 (4%) and lymphocytes=1,782 (18%)<br>- Blood platelets=282,000	- C-Reactive Protein=83mg/L<br>- Globular volume=25%<br>- Hemoglobin=8,6g/dL<br>- Total leukocytes=9,200/band cells=1,932 (21%) and lymphocytes=552 (6%)<br>- Blood platelets=38,000	- C-Reactive Protein=52mg/L<br>- Globular volume=37.5%<br>- Hemoglobin=12.8g/dL<br>- Total leukocytes=4,700/band cells=188 (4%) and lymphocytes=423 (9%)<br>- Blood platelets=112,000	- C-Reactive Protein=301.6mg/L;<br>- Globular volume=31.2%<br>- Hemoglobin=10,7g/dL<br>- Total leukocytes=11,800/band cells=1,770 (15%) and lymphocytes=354 (3%).<br>- Blood platelets=252,000
**Laboratory tests 24 hours before death**	- C-Reactive Protein=201mg/dL;<br>- Globular volume=26.6%<br>- Hemoglobin=9.5g/dL<br>- Leukocytes=15,000/ band cells=2400 (16%) and lymphocytes=1200 (8%);<br>- Blood platelets=239,000	- C-Reactive Protein=133mg/dL;<br>- Globular volume=19.5%<br>- Hemoglobin=7.2g/dL<br>- Leukocytes=50,200/ band cells=3012 (6%) and lymphocytes=3514 (7%).<br>- Blood platelets=318,000	- C-Reactive Protein=139.9mg/dL;<br>- Globular volume=30%<br>- Hemoglobin=10g/dL<br>- Leukocytes=9,900/band cells=693 (7%) and lymphocytes=891 (9%).<br>- Blood platelets=481,000	- C-Reactive Protein=270mg/dL;<br>- Globular volume=23%<br>- Hemoglobin=8.0g/dL<br>- Leukocytes=22,000/band cells=2420 (11%) and lymphocytes=440 (2%).<br>- Blood platelets=356,000	- C-Reactive Protein=407mg/dL;<br>- Globular volume=29.4%<br>- Hemoglobin=9.7g/dL<br>- Leukocytes=9,400/band cells=1,034 (11%) and lymphocytes=1,316 (14%).<br>- Blood platelets=142,000	- C-Reactive Protein=291.5mg/dL;<br>- Globular volume=27.1%<br>- Hemoglobin=9.1g/dL<br>- Leukocytes=24,900/band cells=1,743 (7%) and lymphocytes=498 (2%).<br>- Blood platelets=175,000
**Therapeutic drugs**	- Hydroxychloroquine 800mg/day on the first day and 400mg/day on the other days + Azithromycin 500mg/day for 5 days;<br>- Oseltamivir 150mg/day for 5 days;<br>- Piperacillin + Tazobactam 18mg/day (choice of this antibiotic according to the protocol for the patient's profile)	- Hydroxychloroquine 800mg/day on the first day and 400mg/day on the other days + Azithromycin 500mg/day for 5 days;<br>- Oseltamivir 150mg/day for 5 days;<br>- Ceftriaxone 2g/day (choice of this antibiotic according to the protocol for the patient's profile).	- Piperacillin + tazobactam 18mg/day (choice of this antibiotic according to the protocol for the patient's profile).	- Hydroxychloroquine 800mg/day on the first day and 400mg/day on the other days + Azithromycin 500mg/day for 5 days;<br>- Oseltamivir 150mg/day for 5 days;<br>- Metronidazol 1,5g/day<br>- Meropenem 1g/day,<br>- Linezolid 1,2g/day (choice of these antibiotics according to the diagnosis and protocol for the patient's profile).	- Hydroxychloroquine 800mg/day on the first day and 400mg/day on the other days + Azithromycin 500mg/day for 5 days;<br>- Oseltamivir 150mg/day for 5 days;<br>- Ceftriaxone 2g/day (choice of this antibiotic according to the protocol for the patient's profile).	- Hydroxychloroquine 800mg/day on the first day and 400mg/day on the other days + Azithromycin 500mg/day for 5 days;<br>- Oseltamivir 150mg/day for 5 days;<br>- Meropenem 1g/day, Linezolida 1,2g/day (choice of these antibiotics according to the diagnosis and protocol for the patient's profile).

	CASE 8	CASE 9	CASE 10	CASE 11	CASE 12	CASE 13
**Gender / Age**	Female, 70 years old	Male, 86 years old	Male, 46 years old	Female, 93 years old	Male, 64 years old	Female 75 years old
**Comorbidities**	Type 2 Diabetes Mellitus<br>Systemic Arterial Hypertension<br>Liver transplantation (2013) – viral hepatitis<br>Bladder cancer	Prostate cancer (previous surgical treatment – prostatectomy)<br>Abdominal Aortic Aneurysm<br>Giant Cells Arteritis	Dyslipidemia	Type 2 Diabetes Mellitus<br>Systemic Arterial Hypertension<br>Dyslipidemia<br>Senile dementia<br>Class I Obesity	Systemic Arterial Hypertension	Type 2 Diabetes Mellitus<br>Systemic Arterial Hypertension<br>Coronary Disease<br>Total Atrioventricular Block<br>Heart Failure<br>Chronic Kidney Disease<br>Dyslipidemia<br>Bronchial Asthma/Chronic Obstructive Pulmonary Disease<br>Hypothyroidism
**Computed tomography chest at admission**	Peripheral, multifocal and bilateral "opacities with ground-glass attenuation", thickening of the pulmonary septum, suggestive of viral pulmonary infection. Presence of centrilobular and paraseptal emphysema.<br>Cardiomegaly	Peripheral, multifocal and bilateral "opacities with ground-glass attenuation", thickening of the pulmonary septum, suggestive of viral pulmonary infection.	Peripheral, multifocal and bilateral "opacities with ground-glass attenuation" suggest viral pulmonary infection.	Diffuse and bilateral "opacities with ground-glass attenuation", thickening of the pulmonary septum, suggestive of viral pulmonary infection.	Diffuse and bilateral "opacities with ground-glass attenuation", thickening of the pulmonary septum, suggestive of viral pulmonary infection. Presence of small pleural effusion.	Peripheral, multifocal and bilateral "opacities with ground-glass attenuation", thickening of the pulmonary septum, suggestive of viral pulmonary infection. Presence of pulmonary consolidation, air bronchograms, and small pleural effusion. Presence of centrilobular and parasseptal emphysema. Cardiomegaly
**Relevant initial laboratory tests**	- C-Reactive Protein=16.0 mg/L;<br>- Globular volume=40.4% - Hemoglobin=12.5g/dL<br>- Total leukocytes=7,380/neutrophils=5,850 (80.6%) and lymphocytes=820 (11.1%).<br>- Blood platelets=218.000	- C-Reactive Protein=105.9 mg/L;<br>- Globular volume=32.5%<br>- Hemoglobin=11.5g/dL<br>- Total leukocytes=5,900 / band cells=708 (12%) and lymphocytes=472 (8%).<br>- Blood platelets=110,000	- C-Reactive Protein=155.3 mg/L;<br>- Globular volume=41.9%<br>- Hemoglobin=15.2g/dL<br>- Total leukocytes=4,500/band cells=315 (7%) and lymphocytes=1035 (23%).<br>- Blood platelets=138,000	- C-Reactive Protein=107.5 mg/L;<br>- D-dimer=6,544 μg/mL<br>- Globular volume=27%<br>- Hemoglobin=9.5g/dL<br>- Total leukocytes=7,100/band cells=355 (5%) and lymphocytes=639 (9%).<br>- Blood platelets=111,000	- C-Reactive Protein=420.6 mg/L;<br>- Globular volume=32.4%<br>- Hemoglobin=12.5g/dL<br>- Total leukocytes=9,300/band cells=279 (3%) and lymphocytes=651 (7%).<br>- Blood platelets=354.000/mm^3	- C-Reactive Protein=300.8 mg/L;<br>- Globular volume=30.1%<br>- Hemoglobin=10.7g/dL<br>- Total leukocytes=21,300/band cells=1,278 (6%) and lymphocytes=1,917 (9%).<br>- Blood platelets =205.000/mm^3
**Laboratory tests 24 hours before death**	- C-Reactive Protein=16.0mg/dL;<br>- Globular volume=25.7 %<br>- Hemoglobin=7.6g/dL<br>- Total leukocytes=15,880 and lymphocytes=1260 (7.9%).	- C-Reactive Protein=307.5mg/dL;<br>- Globular volume=25.2%<br>- Hemoglobin=9.0g/dL<br>- Leukocytes=8,900/band cells=445 (5%) and lymphocytes=712 (8%).<br>- Blood platelets=99,000	- C-Reactive Protein=27.5mg/dL;<br>- Globular volume=29%<br>- Hemoglobin=10.2g/dL<br>- Leukocytes=19,500/band cells=1365 (7%) and lymphocytes=975 (5%).<br>- Blood platelets=175,000	- C-Reactive Protein=330mg/dL;<br>- Troponin=14.5 pg/mL<br>- Hemoglobin=8.1g/dL<br>- Leukocytes=15,600/band cells=1092 (7%) and lymphocytes=780 (5%).<br>- Blood platelets=76,000	- C-Reactive Protein=464.6mg/dL;<br>- Globular volume=34.6 %<br>- Hemoglobin=12.3g/dL<br>- Leukocytes=10,600/band cells=424 (4%) and lymphocytes=116 (12%).<br>- Blood platelets=434.000/ mm^3	- C-Reactive Protein=186.7mg/dL;<br>- Globular volume=26.6 %<br>- Hemoglobin=9.3g/dL<br>- Leukocytes=34,900/band cells=6,282 (18%) and lymphocytes=2,094 (6%).<br>- Blood platelets =155.000/mm^3
**Therapeutic drugs**	- Ceftriaxone 2g/day<br>Azithromycin 500mg/day for 5 days; (choice of this antibiotic according to the protocol for the patient's profile)<br>- Oseltamivir 150mg/day for 5 days;<br>- Enoxaparin 1mg/kg/day- full anticoagulation, indicated due to high D-dimers values.<br>- Prednisone 60mg/day.<br>- Piperacillin + Tazobactam 18mg/day (sepsis therapy)	Drugs used in previous admission, one month before:<br>- Hydroxychloroquine 900mg/day on the first day and 450mg/day on the other days + Azithromycin 500mg/day for 5 days;<br>- Oseltamivir 150mg/day for 5 days;<br>Current admission:<br>- Meropenem 1g/day (choice of this antibiotic according to the diagnosis and protocol for the patient's profile)	-Azithromycin 500mg/day for 5 days;<br>Dexamethasone 6mg/day	-Azithromycin 500mg/day for 5 days;<br>-Oseltamivir 150mg/day for 5 days;<br>-Meropenem 1g/day (choice of these antibiotics according to the diagnosis and protocol for the patient's profile)<br>- Enoxaparin 1mg/kg/day- full anticoagulation for the treatment of pulmonary thromboembolism;<br>- Dexamethasone 6mg/day	-Azithromycin 500mg/day for 5 days;<br>-Ceftriaxone 2g/day (choice of this antibiotic according to the protocol for the patient's profile)<br>-Dexamethasone 6mg/day<br>-Enoxaparin 40mg/day	-Azithromycin 500mg/day for 5 days;<br>-Ceftriaxone 2g/day (choice of this antibiotic according to the protocol for the patient's profile)<br>-Dexamethasone 6mg/day<br>-Enoxaparin 20mg/day (prophylactic dose adjusted - Chronic Kidney Disease)<br>- Piperacillin + Tazobactam 18mg/day (sepsis therapy)

	CASE 15	CASE 16	CASE 17	CASE 18	CASE 19	CASE 20
**Gender / Age**	Male, 64 years old	Male, 57 years old	Female, 81 years old	Male, 57 years old	Female, 60 years old	Male, 75 years old
**Comorbidities**	Chronic Non-Dialysis Kidney Disease<br>Kidney Transplant (02 – 1995 and 2011)<br>Systemic Arterial Hypertension<br>Coronary Disease<br>Dyslipidemia<br>Hyperuricemia<br>Chronic Atrial Fibrillation<br>Obstructive Sleep Apnea<br>Benign Prostatic Hyperplasia<br>Nephrolithiasis	Type 2 Diabetes Mellitus<br>Systemic Arterial Hypertension<br>Dyslipidemia<br>Coronary Disease<br>Hepatic Steatosis<br>Class II Obesity	Type 2 Diabetes Mellitus<br>Systemic Arterial Hypertension<br>Dyslipidemia<br>Osteoporosis	Systemic Arterial Hypertension	Left Ventricular uncompressed heart failure<br>Bronchial Asthma<br>Hodgkin's Lymphoma (30 years ago)	Type 2 Diabetes Mellitus<br>Systemic Arterial Hypertension<br>Dyslipidemia<br>Hyperuricemia<br>Coronary Disease (Acute Myocardium Infarction -Apr/2020)
**Computed tomography chest at admission**	Peripheral, multifocal and bilateral "opacities with ground-glass attenuation" suggest viral pulmonary infection.	Peripheral, multifocal and bilateral "opacities with ground-glass attenuation" suggest viral pulmonary infection. Presence of bronchial thickening.	Diffuse and bilateral “opacities with ground-glass attenuation”, thickening of the pulmonary septum, suggestive of viral pulmonary infection.	Diffuse and bilateral “opacities with ground-glass attenuation”, thickening of the pulmonary septum, suggestive of viral pulmonary infection.	Diffuse and bilateral “opacities with ground-glass attenuation”, thickening of the pulmonary septum, suggestive of viral pulmonary infection.<br>Presence of bronchial thickening.<br>Presence of bilateral pleural thickening.<br>The presence of pleural effusion is located on the left.	Diffuse and bilateral “opacities with ground-glass attenuation”, thickening of the pulmonary septum, suggestive of viral pulmonary infection.<br>Presence of bronchial thickening.<br>Presence of diffuse bilateral bronchiectasis.<br>Presence of parasseptal emphysema.
**Relevant initial laboratory tests**	- C-Reactive Protein=66.2 mg/L;<br>- Globular volume=31%<br>- Hemoglobin=11g/dL<br>- Total leukocytes=4,000/band cells=0 (0%) and lymphocytes=960 (24%).<br>- Blood platelets =162.000/mm³	- C-Reactive Protein=154.2 mg/L;<br>- Globular volume=38.3%<br>- Hemoglobin=14g/dL<br>- Total leukocytes=14,500/band cells=1305 (9%) and lymphocytes=435 (3%).<br>- Blood platelets =186.000/mm³	- C-Reactive Protein=199.4 mg/L;<br>- Globular volume=41.3%<br>- Hemoglobin=13.7g/dL<br>- Total leukocytes=22,100/band cells=1326 (6%) and lymphocytes=663 (3%).<br>- Blood platelets =243.000/mm³	- C-Reactive Protein=47.7 mg/L;<br>- Globular volume=43.1%<br>- Hemoglobin=15.8g/dL<br>- Total leukocytes=7,400/band cells=0 (0%) and lymphocytes=1332 (18%).<br>- Blood platelets =146.000/mm³	- C-Reactive Protein=158.6 mg/L;<br>- Globular volume=35.5%<br>- Hemoglobin=11.9g/dL<br>- Total leukocytes=8,100/band cells=81 (1%) and lymphocytes=405 (5%).<br>- Blood platelets =244.000/mm³	- C-Reactive Protein=267mg/L;<br>- Globular volume=43.1%<br>- Hemoglobin=14.9g/dL<br>- Total leukocytes=13,100/band cells=655 (5%) and lymphocytes=917 (7%).<br>- Blood platelets =200.000/mm³
**Laboratory tests 24 hours before death**	- C-Reactive Protein=392.5mg/dL;<br>- Globular volume=26.5%<br>- Hemoglobin=10.7g/dL<br>- Leukocytes=9,800/band cells=686 (7%) and lymphocytes=1470 (15%).<br>- Blood platelets =232.000/mm³	- C-Reactive Protein=267.2mg/dL;<br>- Globular volume=27%<br>- Hemoglobin=9g/dL<br>- Leukocytes=15,300/band cells=459 (3%) and lymphocytes=2448 (16%).<br>- Blood platelets =216.000/mm³	- C-Reactive Protein=16.3mg/dL;<br>- Globular volume=29.2%<br>- Hemoglobin=9.5g/dL<br>- Leukocytes=28,900/band cells=867 (3%) and lymphocytes=1156 (4%).<br>- Blood platelets =141.000/mm³	- C-Reactive Protein=281.6mg/dL;<br>- Globular volume=23.1%<br>- Hemoglobin=7.8g/dL<br>- Leukocytes=15,800/band cells=6004 (38%) and lymphocytes=1264 (8%).<br>- Blood platelets =395.000/mm³	- C-Reactive Protein=264.8mg/dL;<br>- Globular volume=19.7%<br>- Hemoglobin=7.7g/dL<br>- Leukocytes=8,400/band cells=1,260 (15%) and lymphocytes=168 (2%).<br>- Blood platelets =58.000/mm³	- C-Reactive Protein=226.8mg/dL;<br>- Globular volume=19.7%<br>- Hemoglobin=11g/dL<br>- Leukocytes=19,100/band cells=191 (1%) and lymphocytes=573 (3%).<br>- Blood platelets =336.000/mm³
**Therapeutic drugs**	-Azithromycin 500mg/day for 5 days;<br>-Ceftriaxone 2g/day (choice of this antibiotic according to the protocol for the patient's profile)<br>-Piperacillin + Tazobactam 9mg/day (sepsis therapy)<br>-Prednisone 10mg/day<br>-Enoxaparin 20mg/day (prophylactic dose adjusted - Chronic Kidney Disease)	-Azithromycin 500mg/day for 5 days;<br>-Ceftriaxone 2g/day (choice of this antibiotic according to the protocol for the patient's profile)<br>-Dexamethasone 6mg/day<br>- Enoxaparin 40mg/day<br>- Piperacillin + Tazobactam 18mg/day (sepsis therapy)<br>-Alteplase 100mg intravenous for thrombolysis of pulmonary thromboembolism	-Azithromycin 500mg/day for 5 days;<br>-Ceftriaxone 2g/day (choice of this antibiotic according to the protocol for the patient's profile)<br>-Oseltamivir 150mg/day for 5 days;<br>-Dexamethasone 6mg/day<br>-Enoxaparin 40mg/day<br>- Piperacillin + Tazobactam 18mg/day (sepsis therapy)	-Azithromycin 500mg/day for 5 days;<br>-Ceftriaxone 2g/day (choice of this antibiotic according to the protocol for the patient's profile).<br>-Enoxaparin 40mg/day for 5 days and 180mg/day (full anticoagulation) for 7 days. Prophylactic anticoagulation restarted - dose 40mg/day.<br>-Dexamethasone 6mg/day for 10 days;<br>-Daptomycin 1g/day (treatment of subcutaneous tissue infection)<br>-Hydrocortisone 300mg/day (7 days) – bronchospasm treatment<br>-Meropenem 3g/day (sepsis therapy)<br>-Linezolid 1.2g/day (sepsis therapy)<br>-Polymyxin B 4.000.000UI/day (sepsis therapy)	-Azithromycin 500mg/day for 5 days;<br>-Ceftriaxone 2g/day for 5 days (choice of this antibiotic according to the protocol for the patient's profile)<br>-Dexamethasone 6mg/day for 10 days<br>-Enoxaparin 60mg/day<br>-Clopidogrel 75mg/day<br>-Piperacillin + Tazobactam 18mg/day for 5 days (sepsis therapy)<br>-Oxacilin 12g/day (bacterial endocarditis)<br>-Fluconazol 400mg/day (08 days)	-Azithromycin 500mg/day for 5 days;<br>-Ceftriaxone 2g/day for 5 days (choice of this antibiotic according to the protocol for the patient's profile)<br>-Dexamethasone 6mg/day for 10 days<br>-Enoxaparin 80mg/day (full anticoagulation)

	CASE 21	CASE 23	CASE 28	CASE 29	CASE 30	CASE 31
**Gender / Age**	Male, 70 years old	Male, 57 years old	Male, 86 years old	Male, 83 years old	Female, 72 years old	Female, 72 years old
**Comorbidities**	Type 2 Diabetes Mellitus<br>Systemic Arterial Hypertension<br>Atrial Fibrillation<br>Dyslipidemia<br>Class II Obesity<br>Interstitial Pulmonary Fibrosis<br>Previous smoking	Type 2 Diabetes Mellitus<br>Systemic Arterial Hypertension<br>BronchiIal Asthma<br>Class III Obesity	Type 2 Diabetes Mellitus<br>Systemic Arterial Hypertension<br>Dyslipidemia<br>Benign Prostatic Hyperplasia	Systemic Arterial Hypertension<br>Coronary Disease<br>Previous smoking	Type 2 Diabetes Mellitus<br>Systemic Arterial Hypertension<br>Dyslipidemia<br>Hypothyroidism	Systemic Arterial Hypertension<br>Humerus fracture fixation surgery (two weeks before admission)
**Computed tomography chest at admission**	Peripheral, multifocal and bilateral "opacities with ground-glass attenuation" suggest viral pulmonary infection.<br>Interstitial Pulmonary Fibrosis<br>Cardiomegaly	Diffuse and bilateral “opacities with ground-glass attenuation”, thickening of the pulmonary septum, suggestive of viral pulmonary infection.	Diffuse and bilateral “opacities with ground-glass attenuation”, thickening of the pulmonary septum, suggestive of viral pulmonary infection.	Diffuse and bilateral “opacities with ground-glass attenuation”, thickening of the pulmonary septum, suggestive of viral pulmonary infection, with involvement more than 50% of the lung parenchyma.	Diffuse and bilateral “opacities with ground-glass attenuation”, thickening of the pulmonary septum, suggestive of viral pulmonary infection, with involvement more than 50% of the lung parenchyma.	Diffuse and bilateral “opacities with ground-glass attenuation”, thickening of the pulmonary septum, suggestive of viral pulmonary infection, with involvement more than 50% of the lung parenchyma.
**Relevant initial laboratory tests**	- C-Reactive Protein=156.9 mg/L;<br>- Globular volume=52.8%<br>- Hemoglobin=18.2g/dL<br>- Total leukocytes=16,000/band cells=160 (1%) and lymphocytes=320 (2%).<br>- Blood platelets =160.000/mm³	- C-Reactive Protein=47.8 mg/L;<br>- Globular volume=47.8%<br>- Hemoglobin=17.3g/dL<br>- Total leukocytes=7,800/band cells=234 (3%) and lymphocytes=1,248(16%).<br>- Blood platelets =131.000/mm³	- C-Reactive Protein=86 mg/L;<br>- Globular volume=36.9%<br>- Hemoglobin=13.6g/dL<br>- Total leukocytes=5,300/band cells=106 (2%) and lymphocytes=1,060(20%).<br>- Blood platelets =122.000/mm³	- C-Reactive Protein=147.25 mg/L;<br>- Globular volume=38%<br>- Hemoglobin=13.8g/dL<br>- Total leukocytes=9,200/band cells=276 (3%) and lymphocytes=184 (2%).<br>- Blood platelets =190.000/mm³	- C-Reactive Protein=111.6 mg/L;<br>- Globular volume=34.1%<br>- Hemoglobin=11.7g/dL<br>- Total leukocytes=6,000/band cells=360 (6%) and lymphocytes=1,020 (17%).<br>- Blood platelets =222.000/mm³	- C-Reactive Protein=114 mg/L;<br>- Globular volume=30.9%<br>- Hemoglobin=11.6g/dL<br>- Total leukocytes=5,900/band cells=295 (5%) and lymphocytes=472 (8%).<br>- Blood platelets =227.000/mm³
**Laboratory tests 24 hours before death**	- C-Reactive Protein=8.7mg/dL;<br>- Globular volume=32%<br>- Hemoglobin=11.3g/dL<br>- Leukocytes=10,500/band cells=525 (5%) and lymphocytes=420 (4%).<br>- Blood platelets =67.000/mm³	-C-Reactive Protein=236.2mg/dL;<br>- Globular volume=26.6%<br>- Hemoglobin=9.3g/dL<br>- Leukocytes=25,000/band cells=4,000 (16%) and lymphocytes=2,000 (8%).<br>- Blood platelets =132.000/mm³	- C-Reactive Protein=180mg/dL;<br>- Globular volume=39.5%<br>- Hemoglobin=14.1g/dL<br>- Leukocytes=4,700/band cells=141 (3%) and lymphocytes=282 (6%).<br>- Blood platelets =132.000/mm³	- C-Reactive Protein=30.7mg/dL;<br>- Globular volume=38.4%<br>- Hemoglobin=13.4g/dL<br>- Leukocytes=23,300/band cells=2,097 (9%) and lymphocytes=233 (1%).<br>- Blood platelets =436.000/mm³	- C-Reactive Protein=339.9mg/dL;<br>- Globular volume=23.7%<br>- Hemoglobin=8.5g/dL<br>- Leukocytes=32,200/band cells=7,084 (22%) and lymphocytes=1,288 (4%).<br>- Blood platelets =190.000/mm³	- C-Reactive Protein=53.9mg/dL;<br>- Globular volume=21.2%<br>- Hemoglobin=7.7g/dL<br>- Leukocytes=7,900/band cells=474 (6%) and lymphocytes=474 (6%).<br>- Blood platelets =180.000/mm³
**Therapeutic drugs**	- Due to laboratory coagulation disorders, anticoagulants were not administered.<br>-Ceftriaxone 2g/day (choice of this antibiotic according to the protocol for the patient's profile)<br>- Azithromycin 500mg/day for 5 days;<br>- Tocilizumab 300mg/day for two days;<br>- Methylprednisolone 125mg/day for ten days;<br>- Piperacillin + Tazobactam 18mg/day (sepsis therapy)<br>-Enoxaparin 60mg/day (full anticoagulation) for 12 days.<br>- Anidulafungin 200mg/day (1 day);<br>- Meropenen 3g/day (2 days);<br>- Linezolid 1.2g/day (2 days);<br>- Polymyxin B Sulfate 1.000.000UI (1 day)	-Azithromycin 500mg/day for 5 days;<br>-Ceftriaxone 2g/day (choice of this antibiotic according to the protocol for the patient's profile);<br>- Oseltamivir 150mg/day for 5 days;<br>-Dexamethasone 6mg/day for 5 days;<br>- Enoxaparin 40mg/day;<br>- Enoxaparin 60mg/day a partir de 23/07;<br>- Piperacillin + Tazobactam 18mg/day for 7 days (sepsis therapy);<br>- Hidrocortisona 100mg/day;<br>- Meropenem 3g/day (for 9 days);<br>- Linezolida 1.2g/day (for 9 days);<br>- Sulfato de Polimixina B 3.000.000UI (for 5 days)	Azithromycin 500mg/day for 5 days;<br>-Ceftriaxone 2g/day (choice of this antibiotic according to the protocol for the patient's profile);<br>-Dexamethasone 6mg/day for 5 days;<br>- Enoxaparin 40mg/day;<br>- Methylprednisolone 125mg/day for 03 days	-Azithromycin 500mg/day for 5 days;<br>-Ceftriaxone 2g/day (choice of this antibiotic according to the protocol for the patient's profile);<br>-Dexamethasone 6mg/day for 10 days;<br>- Hydrocortisone 200mg/day;<br>- Enoxaparin 60mg/day for 9 days;<br>- Enoxaparin 160mg/day for seven days;<br>- Cefepime 6g/day<br>- Meropenen 3g/day (2 days);<br>-Ampicillin+Sulbactam 12g/day<br>-Linezolid 1.2g/day (7 days)	-Oseltamivir 150mg/day for 1 day;<br>Dexamethasone 6mg/day for 10 days;<br>- Hydrocortisone 200mg/day 20 days;<br>- Enoxaparin 40mg/day;<br>-Azithromycin 500mg/day for 5 days;<br>-Ceftriaxone 2g/day (choice of this antibiotic according to the protocol for the patient's profile);<br>- Piperacillin + Tazobactam 18mg/day (sepsis therapy) for 8 days<br>- Moxifloxacin 400mg/day 1 day	-Oseltamivir 150mg/day for 1 day;<br>Dexamethasone 6mg/day for 10 days;<br>- Hydrocortisone 200mg/day 13 days;<br>Azithromycin 500mg/day for 5 days;<br>-Ceftriaxone 2g/day (choice of this antibiotic according to the protocol for the patient's profile);<br>- Meropenen 3g/day (10 days);<br>- Polymyxin B Sulfate 1.000.000UI (1 day)<br>-Linezolid 1.2g/day (7 days);<br>- Enoxaparin 40mg/day for 19 days;<br>- Enoxaparin 120mg/day

Supplementary Table S3: Allelic frequency analysis between COVID-19 and H1N1 groups for *PRF1* gene.

**reference SNP†<br>Allele variation [1/2]**	**Allele 1**	**Allele 2**	**MAF***	
**rs10999426 [G/A]**	G	A	TOPMED	1000G
COVID-19	31 (64.6)	17 (35.4)	A=0.2176	A=0.1404
H1N1	16 (80.0)	4 (20.0)		
**rs885821 [G/A]**	G	A		
COVID-19	39 (81.3)	9 (18.7)	A=0.1409	A=0.1126
H1N1	16 (80.0)	4 (20.0)		
***rs*885822 [G/A]**	G	A		
COVID-19	21 (56.8)	16 (43.2)	G=0.3267	G=0.3041
H1N1	4 (20.0)	16 (80.0)		
**rs35947132 [G/A]**	G	A		
COVID-19	44 (91.6)	4 (8.4)	A=0.0291	A=0.0132
H1N1	20 (100.0)	0 (0.0)		

†SNP identifier based on NCBI dbSNP; Allele 1=wild; Allele 2=polymorphic; *MAF=Minimum Allele Frequency from NCBI SNP for TOPMED and 1000G data base; Allele frequency was expressed by absolute number and frequency.
